# NrnC, an RNase D-Like Protein From *Agrobacterium*, Is a Novel Octameric Nuclease That Specifically Degrades dsDNA but Leaves dsRNA Intact

**DOI:** 10.3389/fmicb.2018.03230

**Published:** 2019-01-07

**Authors:** Zenglin Yuan, Fei Gao, Kun Yin, Lichuan Gu

**Affiliations:** ^1^State Key Laboratory of Microbial Technology, Shandong University, Jinan, China; ^2^Shandong Institute of Parasitic Diseases, Shandong Academy of Medical Sciences, Jining, China

**Keywords:** nuclease, DEDDy, RNase D, NrnC, octamer

## Abstract

NrnC from *Agrobacterium tumefaciens* (At_NrnC, UniProt accession number A9CG28) is a nuclease containing a single DEDDy domain. Here, we determined the structures of both the apo and metal-ion-bound forms of At_NrnC. Although the overall structure of the At_NrnC protomer is similar to that of the RNase D exonuclease domain, nuclease assays unexpectedly revealed that At_NrnC possesses remarkably different substrate specificity. In contrast to RNase D, which degrades both single-stranded RNA (ssRNA) and double-stranded RNA (dsRNA), At_NrnC hydrolyses ssRNA, single-stranded DNA (ssDNA), and double-stranded DNA (dsDNA) with high efficiency but does not degrade dsRNA. Crystal packing analysis and biochemical data indicated that At_NrnC forms an octameric hollow cylindrical structure that allows ssRNA, ssDNA, and dsDNA, but not dsRNA, to enter the central tunnel where the multiple active sites perform hydrolysis. This novel structural feature confers a high processivity and is responsible for the preference of At_NrnC for longer dsDNA substrates.

## Introduction

Nucleases are a highly diverse group of enzymes that cleave the phosphodiester bonds of nucleic acids. For these proteins, the correlation between catalytic mechanism and biological function is weak (Yang, 2011). A single bacterial cell often contains dozens of nucleases, which play important roles in numerous metabolic pathways, where one enzyme may degrade multiple substrates and one substrate may be cleaved by multiple enzymes. These features render it challenging to identify the natural substrate(s) and biological role of a particular nuclease.

**FIGURE 1:**
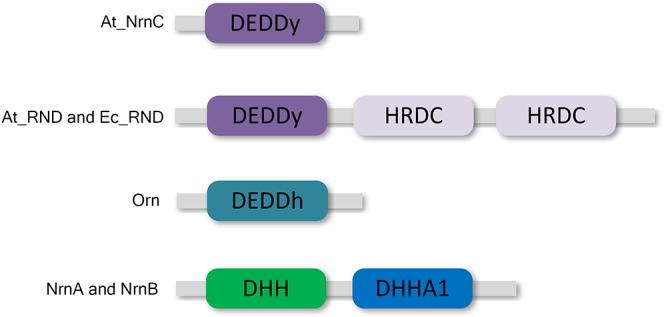
Domain organisation of At_NrnC and other DEDD exonucleases. At_NrnC consists of a single DEDDy domain, whereas Orn contains a single DEDDh domain. RNase D from *Escherichia coli* (Ec_RND) and its close homologue from *Agrobacterium fabrum* (At_RND) consist of a DEDDy domain and two additional HRDC domains. NrnA and NrnB contain DHH and DHHA1 domains.

DEDD 3′–5′ exonucleases are widely distributed among both prokaryotes and eukaryotes (Zuo and Deutscher, 2001). All DEDD exonucleases contain a conserved DEDD motif and can be divided into two subgroups, DEDDh and DEDDy, based on whether they contain a histidine or a tyrosine located four or five residues before the final aspartic acid of the DEDD motif. DEDD exonucleases utilise a mechanism involving two metal ions to hydrolyse RNA or DNA. The divalent metal ions Mg^2+^ and Mn^2+^ are common cofactors for DEDD exonucleases. A typical example of DEDDy exonucleases, which are found in many bacteria, is RNase D (Figure 1). RNase D from *Escherichia coli* (Ec_RND) contains two helicase and RNase D C-terminal (HRDC) domains at its C-terminus. It has been proposed that these HRDC domains may participate in substrate binding and contribute to the processivity of RNase D (Zuo et al., 2005).

However, HRDC domains are not highly conserved. Many bacteria contain RNase D-like proteins lacking one or both of the HRDC domains. These truncated RNase D-like proteins have been reported in numerous Alphaproteobacteria. The *Agrobacterium tumefaciens* genome contains two homologues of RNase D, Atu1151 (denoted At_RND, 30% similarity with full-length Ec_RND) and Atu4108 (denoted At_NrnC, 31% similarity with the N-terminal 184 residues of Ec_RND). At_RND contains an exonuclease domain and two HRDC domains, whereas At_NrnC contains a single DEDDy domain. The At_RND exonuclease domain and At_NrnC possess a similarity of 32%. However, most of the studies in this field focused on the full-length RNase D, and it remains uncertain whether the truncated RNase D-like proteins function in a similar manner.

Recently, one truncated RNase D-like protein, NrnC from *Bartonella henselae* (Bh_NrnC, 69% similarity with At_NrnC), was identified as a functional analogue of oligoribonuclease (Orn) from *E. coli* (Ghosh and Deutscher, 1999; Liu et al., 2012). As an essential *E. coli* protein, Orn is a crucial component of the mRNA decay pathway that is required to complete the degradation of mRNA to mononucleotides. Notably, Orn was also suggested to be the primary enzyme responsible for removing 5′-phosphoguanylyl-(3′,5′)-guanosine (pGpG), the final step in the (3′–5′)-cyclic diguanosine monophosphate (c-di-GMP) degradation pathway (Cohen et al., 2015; Orr et al., 2015). In addition to Bh_NrnC, it has been reported that nanoRNases NrnA and NrnB from *Bacillus subtilis*, which belong to the DHH/DHHA1 protein family, are able to complement a conditional promoter mutant of *E. coli orn* (Mechold et al., 2007; Fang et al., 2009). Both *B. henselae* and *B. subtilis* do not possess an Orn homologue.

To elucidate the function and mechanism of truncated RNase D-like proteins, we characterised At_NrnC, a close homologue of Bh_NrnC, using a combination of biochemical and structural biology approaches. At_NrnC forms an octameric hollow cylindrical structure, which allows dsDNA, ssDNA, and ssRNA to enter the central tunnel where the multiple active sites perform hydrolysis. This novel structural feature accounts for the high processivity of At_NrnC and its preference for longer dsDNA substrates despite the lack of HRDC domains.

## Results

### Overall Structure of At_NrnC

**FIGURE 2:**
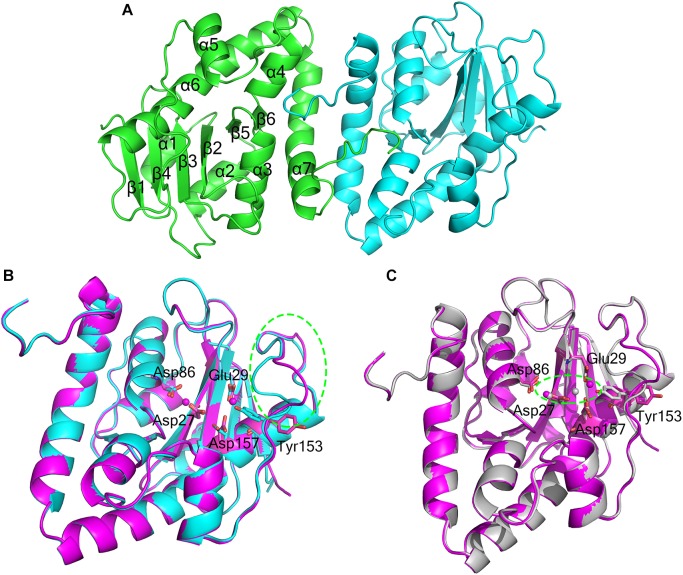
Overall structures of apo-At_NrnC and Mn^2+^-bound At_NrnC. **(A)** Cartoon representation of the structure of At_NrnC in an asymmetric unit; the two individual At_NrnC molecules are shown in green and cyan. In our structure, each At_NrnC protomer contains seven α helices and six β strands. The six β strands are situated in the centre of the protein surrounded by the seven α helices and loops. **(B)** Superposition of apo-At_NrnC and the inactive Mn^2+^-bound At_NrnC, which are shown as cartoon representations in cyan and magenta, respectively. The conserved catalytic DEDDy residues and the Mn^2+^ ions are shown as sticks and spheres, respectively. The outward flipping of Tyr153 and the loop consisting of Lys134 to Ser138 is indicated by the green dashed circle. **(C)** Superposition of the active and inactive forms of Mn^2+^-bound At_NrnC, which are shown as cartoon representations in grey and magenta, respectively. The metal ions are indicated by the green dashed circle. In the structure of the active state, Tyr153 is situated at the typical catalytic site for DEDDy enzymes and the distance between the two Mn^2+^ ions is shorter.

The crystal structure of apo-At_NrnC was determined at 1.5 Å resolution and those of two forms of At_NrnC-Mn^2+^ were determined at 2.5 Å and 2.3 Å resolution. Attempts to obtain crystals of At_NrnC with other metal ions or the product deoxynucleoside monophosphates (dNMPs) through soaking or co-crystallisation were unsuccessful. The apo and metal-bound structures were almost identical and only minor differences were observed (Figures 2B,C). The final model of Atu4108 contained two protein molecules in the asymmetric unit. The At_NrnC protomer consisted of seven α helices and six β strands (Figure 2A). The β-sheet region comprised all six β strands and extended from the surface to the central core. The remaining seven α helices and loops surrounded the above-mentioned strands and made contact with the other molecule of the asymmetric unit. The C-terminal loop extended into a pocket in the second molecule formed from the α3, α4, and α7 helices and the β6 strand. The α7 helix was slightly bent in the middle to better accommodate the C-terminal loop.

### Active Site Structure of At_NrnC

Metal binding was found to exert only a minor influence on the overall structure of At_NrnC. Based on the arrangement registered on the active pocket, we considered one of the metal-bound structures to represent an inactive state and the other an active state (Figures 2B,C). For the inactive state, a short loop from Lys134 to Ser138 near the active pocket was found to flip out, thereby enlarging the active pocket (Figure 2B and Supplementary Figure S1). The key residue Tyr153 also flipped out, which is rare among DEDDy protein structures. Our hypothesis is that this flipping provides space to accommodate the metal ions and Tyr153 flips back to a suitable position before the reaction occurs. In addition, nuclease assays indicated that Tyr153 is essential for At_NrnC function. Indeed, in the structure of the active state, Tyr153 was located at the typical catalytic site for DEDDy enzymes (Figure 2C). In the active pocket, Asp27 and Asp86 coordinated one Mn^2+^, while Glu29 and Asp157 coordinated the other Mn^2+^. The distance between the two Mn^2+^ ions was 6.5 Å in the inactive state and 3.9 Å in the active state (Figure 2C). The former distance of 6.5 Å is considerably longer than the distance of approximately 4 Å reported for other two-metal-ion-dependent nucleases (Yang et al., 2006).

### Crystal Packing and Oligomeric State of At_NrnC

**FIGURE 3:**
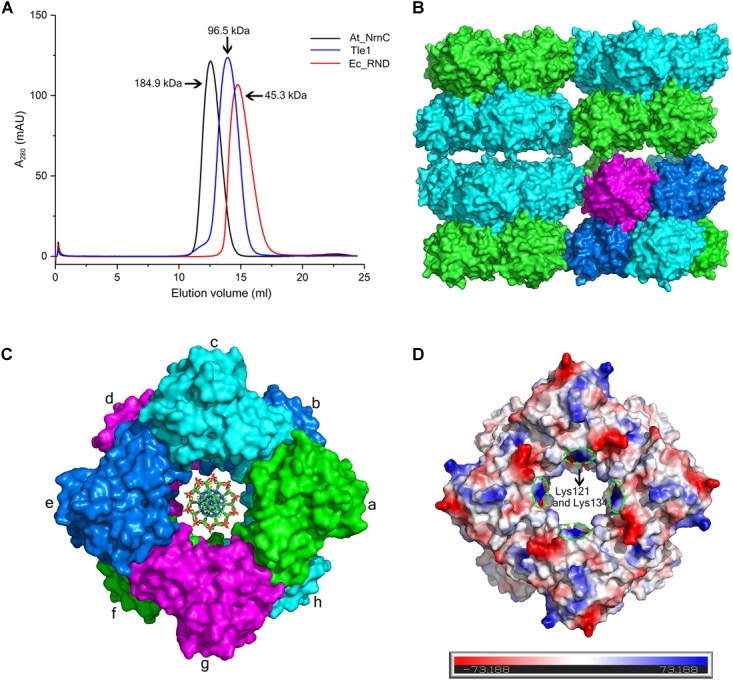
At_NrnC forms an octameric architecture. **(A)** Size-exclusion chromatograms for At_NrnC, Ec_RND, and Tle1, which exhibited elution volumes of 12.55 mL, 14.75 mL, and 13.95 mL, respectively. At_NrnC is predicted to be an octamer with a molecular weight of approximately 200 kDa. **(B)** Crystal packing analysis for At_NrnC. The four asymmetric units in the octamer form an interconnected higher-level cylindrical architecture and in crystal lattice, these cylindrical architectures connect to each other, forming a higher-level architecture. The octamer contains two layers and the two protomers in each asymmetric unit are separated into these two layers (green and cyan). **(C)** Surface representation of the At_NrnC octamer and the manually modelled DNA fragment. In the octamer, each protomer is in contact with four other protomers. For example, protomer a is in contact with protomers b, c, g, and h, sharing buried surface areas of 368.5 Å^2^ with protomer b, 429.6 A^2^ with protomer c, 431.7 Å^2^ with protomer g, and 1296.9 Å^2^ with protomer h. **(D)** Molecular surface of the At_NrnC octamer. The surface colour indicates the local electrostatic potential, which was determined using PyMOL. The green dashed circles indicate the positively charged areas where Lys121 and Lys134 are located.

Size-exclusion chromatography (SEC) of At_NrnC revealed a smaller elution volume than that expected based on its molecular weight, 23 kDa (Figure 3A). The peak position remained the same after rising the NaCl concentration from 100 to 500 mM (data not shown). At_NrnC was then compared with several previously reported proteins (Figure 3A and Supplementary Figure S2). Ec_RND is a 45.3 kDa monomer and its elution volume was 14.75 mL; Tle1 from *Pseudomonas aeruginosa* is a 96.5 kDa monomer and its elution volume was 13.95 mL. The 12.55 mL elution volume of At_NrnC indicated that this protein exists as an octamer with an estimated molecular weight of approximately 200 kDa. To further evaluate the oligomeric state of At_NrnC, analytical ultracentrifugation (AUC) analysis was performed (Supplementary Figure S3). The AUC results indicated that At_NrnC has an estimated molecular weight of approximately 190 kDa in solution, and the addition of Mn^2+^ exerted only a minor influence.

Crystal packing analysis also revealed an octameric structure formed by eight At_NrnC protomers (Figures 3B,C). The overall structure of the octamer is a hollow cylinder composed of two layers with each layer containing four At_NrnC protomers. Each protomer was found to be in contact with four other protomers and the total accessible surface area of the octamer was 64042.7 Å^2^, while the buried surface area between the eight protomers was 20290.9 Å^2^ (Krissinel, 2015). The extensive assembling interface suggests that At_NrnC functions as a genuine octamer in solution. Strong hydrophobic interactions, mainly contributed by the hydrophobic residues of the α7 helix, were present at the interface of the dimer in the asymmetric unit. A large number of acidic and basic residues were observed at the other three interfaces. Therefore, both hydrophobic and electrostatic interactions facilitated the formation of the highly stable octamer.

Furthermore, the sedimentation coefficients were calculated using HYDROPRO (Ortega et al., 2011). The calculated sedimentation coefficients for the proposed octamer, single-layer tetramer, and dimer were 8.69, 5.30, and 3.50 S, respectively. The value derived from the AUC data (8.41 S, ca. 200 kDa) was very close to the calculated value for the proposed octamer (8.69 S, 180 kDa). Taken together, the AUC, SEC, and crystal packing analysis results indicate that the proposed octamer possesses a high stability.

Notably, the At_NrnC octamer features a large tunnel running through the entire complex along the central axis. The dimensions of this pore were analysed using the HOLE programme (Smart et al., 1996), revealing a diameter of 20 Å at the narrowest part (Supplementary Figure S4). The openings of the active sites of the eight protomers are situated on the tunnel wall. These structural features strongly suggest that the nucleic acid substrates are able to thread through the tunnel and undergo hydrolysis inside, which may account for the high processivity and activity of At_NrnC.

### Nuclease Activity of At_NrnC

**FIGURE 4:**
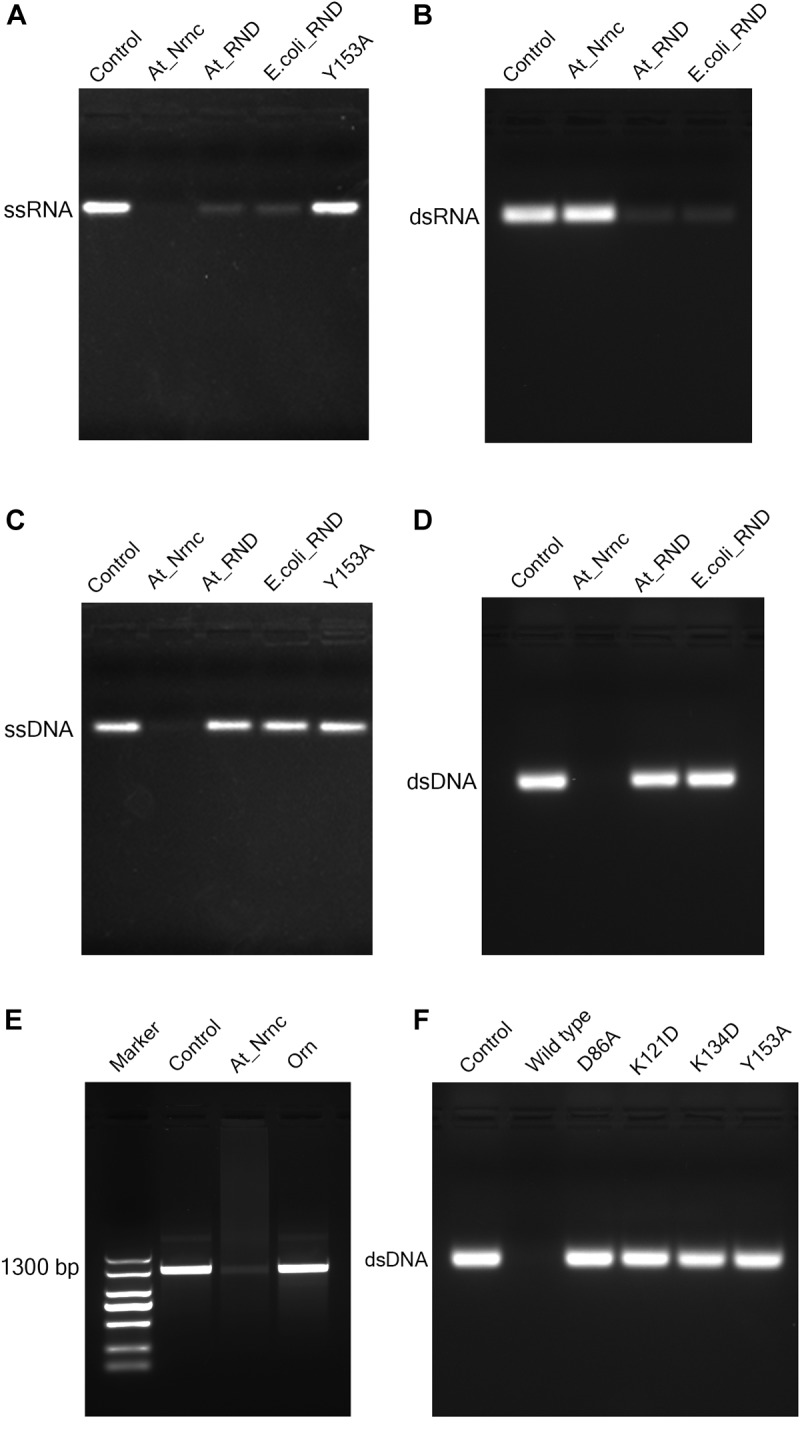
Nuclease activity assays. **(A)** Exonuclease activities of various nucleases toward 33-bp ssRNA. While the two RNase D enzymes degraded almost all of the ssRNA substrate, At_NrnC degraded it completely. **(B)** Exonuclease activities of various nucleases toward 33-bp dsRNA. At_NrnC exhibited no activity toward dsRNA in our assay, whereas the two RNase D enzymes again degraded almost all of the dsRNA substrate. **(C)** Exonuclease activities of various nucleases toward 33-bp ssDNA. At_NrnC degraded ssDNA completely, whereas the two RNase D enzymes exhibited no detectable activity. **(D)** Exonuclease activities of various nucleases toward 33-bp dsDNA. At_NrnC degraded dsDNA completely, whereas the two RNase D enzymes exhibited no detectable activity. **(E)** Exonuclease activities of At_NrnC and Orn toward PCR-amplified 1300-bp DNA. At_NrnC degraded most of the 1300-bp DNA substrate, whereas the activity of Orn was barely detectable. **(F)** Exonuclease activities of At_NrnC and its mutants toward 33-bp dsDNA. The D86A, K121D, K134D, and Y153A mutations all deprived At_NrnC of activity.

RNase D is a 3′ to 5′ hydrolytic exoribonuclease belonging to the DEDDy family. The five key amino acid residues of the DEDDy motif are well conserved among Ec_RND, At_RND, and At_NrnC. It was reported that Ec_RND hydrolyses tRNA substrates and Bh_NrnC, a close homologue of At_NrnC, efficiently hydrolyses small RNA substrates (Cudny et al., 1981; Liu et al., 2012). However, the activities of RNase D and Bh_NrnC proteins toward the different types of nucleic acids have not yet been comprehensively evaluated. Here, the activities of Ec_RND, At_RND, and At_NrnC toward ssRNA, dsRNA, ssDNA, and dsDNA were systematically determined (Figure 4).

Surprisingly, although these three nucleases share the same catalytic domain, they exhibited distinct catalytic activities. Both Ec_RND and At_RND efficiently hydrolysed the ssRNA and dsRNA substrates but exhibited almost no activity toward ssDNA or dsDNA. Unexpectedly, At_NrnC degraded ssRNA but not dsRNA. Moreover, it exhibited high activity toward both ssDNA and dsDNA. From this experiment, the turnover of the 33-mer substrates into monomers catalysed by At_NrnC was roughly estimated as at least 2.67 pmol/μg/min, which is close to that reported for Bh_NrnC (Liu et al., 2012). Next, the activity toward a 1300-bp amplified dsDNA was evaluated and compared with that of Orn, which strongly prefers short oligonucleotides. The results revealed that At_NrnC, but not Orn, efficiently hydrolyses long dsDNA (Figure 4E).

## Discussion

### Structure–Function Implications for Truncated DEDD Exonucleases

**FIGURE 5:**
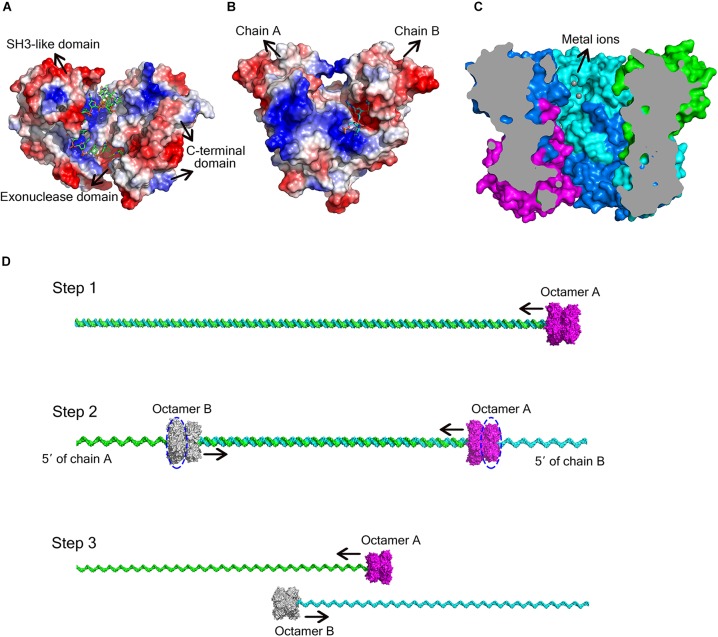
Different ways for DEDD exonucleases to achieve activity and processivity. **(A)** Molecular surface of *E. coli* DNA exonuclease I (PDB code: 4JRP), coloured by local electrostatic potential using PyMOL. DNA exonuclease I possesses three domains: an exonuclease domain (DEDDh), an SH3-like domain, and a C-terminal domain. The latter two domains help to bind and anchor the substrate. **(B)** Molecular surface of *E. coli* RNase T homodimer (PDB code: 3V9Z), coloured by local electrostatic potential using PyMOL. When the two protomers bind to each other, each protomer has a basic region opposite the DEDD catalytic centre of the other protomer, thereby supplying the substrate-binding site for the latter. **(C)** Sliced view of the At_NrnC octamer. The bound Mn^2+^ ions are shown as grey spheres. **(D)** Model for the hydrolysis of dsDNA by At_NrnC. The blue dashed circles indicate the active layers. Two octamers move toward each other on the same dsDNA, with each digesting one chain from the 3′ end while leaving the other chain intact. When the two octamers approach one another, the two chains of the dsDNA separate and each octamer continues to hydrolyse its ssDNA fragment.

DEDD exonucleases participate in numerous nucleic acid metabolism pathways and are widely distributed in bacteria. Since the DEDD motif only supplies the binding site for the metal cofactor, additional components that bind the nucleic acid substrate are typically required for enzymatic function. Many DEDD exonucleases contain DEDD domains connected to other domains. For example, the DEDD domain from the Klenow fragment exhibits no detectable exonuclease activity after removing the polymerase domain (Joyce and Steitz, 1994). Ec_RND has two HRDC domains at its C-terminus, which induce the ring conformation. This domain architecture was also reported for *E. coli* DNA exonuclease I, another member of the DEDD family of exonucleases (Figure 5A; Zuo et al., 2005; Korada et al., 2013). In addition to activation by these non-homologous domains, some DEDD exonucleases can form homodimers that display strong nuclease activity. For example, in the RNase T homodimer from *E. coli*, each protomer contains a basic region situated opposite the DEDD catalytic centre of the other protomer, thereby supplying the substrate-binding site for the latter (Figure 5B; Zuo et al., 2007).

As At_NrnC is a single-DEDDy-domain protein, we suggest that its activity relies on the formation of an octamer (Figure 3). Since the DEDDy catalytic motif of each protomer is located in the central tunnel of the cylindrical octamer, it is readily apparent that the DNA or RNA substrates must enter the central tunnel prior to reaching one of the catalytic centres and undergoing degradation (Figure 5C). The narrowest region of the tunnel possesses a diameter of 20 Å, which is just sufficient for typical B-type dsDNA to pass through but too small for dsRNA (Figure 2C and Supplementary Figure S4). This also raises the question of which layer of active sites is occupied first, since the tunnel contains eight active sites organised into two layers. Structural analysis suggests that, owing to steric hindrance, the DNA is unlikely to curl into the DEDDy catalytic centres of the first layer upon entering the tunnel. Most probably, the DNA (or ssRNA) will pass the first layer and encounter a DEDDy catalytic centre in the second layer (active layer), where the catalysis occurs. This also suggests that two At_NrnC octamers may hydrolyse a dsDNA substrate from both ends simultaneously while moving toward each other on the same dsDNA, with each octamer digesting one chain from the 3′ end and leaving the other chain intact (Figure 5D). When the two octamers approach one another, the two chains of the dsDNA will separate and each octamer can continue to hydrolyse its respective ssDNA fragment to the end. Moreover, owing to the helical nature of dsDNA, At_NrnC has to rotate on its axis while sliding along the dsDNA substrate during catalysis.

**FIGURE 6:**
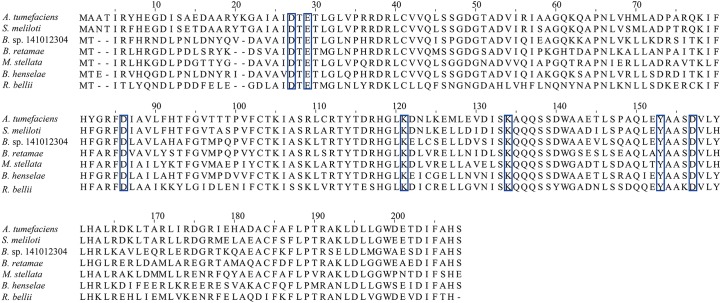
Multiple sequence alignment of At_NrnC with several other truncated RNase D-like proteins from Alphaproteobacteria. The residues comprising the conserved DEDDy motif and the basic amino acids surrounding the tunnel entrance Lys121 and Lys134 are indicated by blue boxes. The identities between At_NrnC and the other truncated RNase D-like proteins range from 50 to 80%.

To test our hypothesis, the distribution of charged residues on the wall of the tunnel was analysed in detail. Large positively charged areas were not observed except for four symmetry-related positively charged residue clusters located on each layer (Figure 3D). Each positively charged residue cluster consisted of Arg117, Lys121, and Lys134. Multiple sequence alignment of At_NrnC with several other truncated RNase D-like proteins from Alphaproteobacteria revealed that Lys121 and Lys134 were more conserved than Arg117 (Figure 6). The importance of these three residues for the nuclease activity of At_NrnC was next examined. The mutations occurring at the expense of Arg117 always resulted in an unstable At_NrnC mutant protein and therefore these mutants could not be tested. The mutations K121D and K134D almost completely abolished the exonuclease activity toward dsDNA (Figure 4F). Gel filtration analysis revealed that both K121D and K134D mutants eluted at the same volume as the wild-type At_NrnC (data not shown), which indicated that these mutations did not affect the oligomeric state. Our explanation is that the Lys121 and Lys134 residues are involved in binding the nucleic acid and guiding it into the central tunnel; consequently, the K121D and K134D mutations would prevent the entry of the nucleic acid and thereby decrease the enzymatic activity.

### Mechanistic Insights Into the Detailed Catalytic Process of the At_NrnC Protomer

**FIGURE 7:**
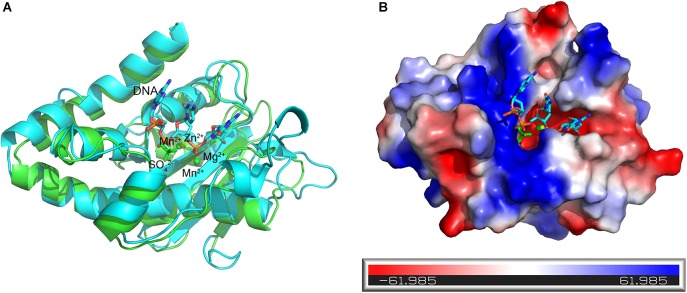
Superposition of the active Mn^2+^-bound At_NrnC and the Klenow fragment–DNA complex structure. **(A)** Cartoon representation of the Mn^2+^-bound At_NrnC (cyan) and the Klenow fragment–DNA complex (green). For clarity, other domains in the Klenow fragment structure were deleted and only the nuclease domain and part of the DNA (in stick form) are shown. The bound metal ions are shown as spheres. **(B)** Molecular surface of At_NrnC, coloured by local electrostatic potential using PyMOL. The bound SO_4_^2-^ and the DNA fragment from the superposition with the Klenow fragment are shown as sticks. While the centre of the active pocket is negatively charged, the left side of the active pocket is positively charged.

The DNA substrate that enters the tunnel has to reach the active site of a protomer to be degraded. To elucidate how the DNA substrate enters the active site of the At_NrnC protomer, we aligned the structure of the active Mn^2+^-bound At_NrnC complex with that of the Klenow fragment–DNA complex (PDB code: 1KFS). For clarity, only the nuclease domain and part of DNA in the Klenow fragment structure are shown (Figure 7A). After structural superposition, At_NrnC and the nuclease domain of the Klenow fragment aligned well and the DNA fragment from the Klenow fragment structure was able to fit into the active pocket of At_NrnC without clash. According to the superposition, the two Mn^2+^ ions were located on two sides of the DNA backbone, which is similar to the case of Mg^2+^ and Zn^2+^ in the Klenow fragment structure (Figure 7A). In addition, we found that a SO_4_^2-^ ion present in the At_NrnC structure was located in the vicinity of a PO_4_^3-^ in the DNA backbone. Analysis of the molecular surface of At_NrnC revealed that the left region of At_NrnC adjacent of the DNA fragment was positively charged, where we suggest that the substrate of At_NrnC binds (Figure 7B). When we observe the active pocket for hydrolysis in the octamer model, the DNA will enter through the left region and then proceed to the catalytic centre, which is consistent with the results of our analysis for the At_NrnC protomer.

At present, the biological role of At_NrnC remains unclear. Although At_NrnC displays high levels of enzymatic activity toward ssDNA, dsDNA, and ssRNA, in our experiments the overexpression of At_NrnC did not affect the growth rate of the host. Like Orn, Bh_NrnC exhibits RNA oligonucleotide degradation activity and was able to complement a conditional *orn* mutant in *E. coli* (Mechold et al., 2006). Although it has been shown that Orn is active toward a 5-mer DNA oligonucleotide, it exhibited no detectable DNase activity toward double-stranded T7 DNA (Niyogi and Datta, 1975; Mechold et al., 2006) or our PCR-amplified 1300-bp fragment (Figure 4E). However, At_NrnC, with its octameric cylindrical architecture, may prefer longer double-stranded DNA. Compared with the ring-like (single-layer) structure, the hollow cylindrical (double-layer) architecture may be more advantageous with respect to ssDNA or ssRNA substrates, since the hollow cylindrical (double-layer) architecture could permit more interactions with the central tunnel. Moreover, dsDNA must be unwound for the degradation to occur and the octamer needs to move along the substrate to perform processive hydrolysis. It was reported that the energy released from the hydrolytic nuclease reaction can power the unwinding of dsRNA (Lee et al., 2012). In our case, it is possible that the energy released from nucleic acid hydrolysis pushes At_NrnC toward the 5′ end of the substrate and assists in the unwinding of the dsDNA.

Owing to its distinct structural features, At_NrnC is a versatile enzyme that can degrade ssDNA, dsDNA, and ssRNA but not dsRNA. Furthermore, as an exonuclease, At_NrnC can degrade its substrate into dNMPs or NMPs, which can be removed easily. Consequently, At_NrnC could be used to remove ssDNA, dsDNA, and ssRNA from a mixture without affecting the recovery of dsRNA.

## Materials and Methods

### Cloning, Expression, and Purification

The *At_NrnC* gene was amplified from genomic DNA of *A. tumefaciens* str. C58. The amplified PCR product was digested with *Bam*HI and *Xho*I and then ligated into a modified pET-15b vector. *E. coli* BL21(DE3) cells harbouring the plasmid of the recombinant At_NrnC protein were grown in LB broth medium containing 100 μg/mL ampicillin at 37°C. Once the bacterial density had reached an OD_600_ of 0.6, the growing temperature was decreased to 16°C and isopropyl β-D-1-thiogalactopyranoside (IPTG) was added to a final concentration of 1 mM to induce expression of the recombinant protein.

After 16 h of induction, the cells were harvested by centrifugation at 5000 × *g* for 20 min. The harvested cells were resuspended in lysis buffer (25 mM Tris–HCl buffer pH 8.0, 100 mM NaCl) and lysed by sonication. To obtain the soluble fraction, the cell lysate was centrifuged at 25000 × *g* for 50 min. The supernatant was then loaded onto a Ni-chelating Sepharose (GE Healthcare) affinity column pre-equilibrated with lysis buffer. The affinity column was washed and resuspended with buffer (25 mM Tris–HCl pH 8.0, 100 mM NaCl, 10 mM imidazole). The 6 × His tag was removed using rhinovirus 3C protease. The At_NrnC protein was then eluted using elution buffer (25 mM Tris–HCl pH 8.0, 100 mM NaCl). The eluate was then loaded onto a Source 15Q anion-exchange column (GE Healthcare) and eluted using a 200 mL linear gradient of 0–0.5 M NaCl. Then, SEC was performed using a Superdex 200 column (GE Healthcare) equilibrated with 25 mM Tris–HCl pH 8.0 containing 100 mM NaCl. The fractions containing At_NrnC were collected according to the protein purity as determined by SDS–PAGE, and the final protein concentration used for crystallisation was 10 mg/mL.

### Oligomeric State Analysis

For SEC assays, the purified protein samples were loaded onto a Superdex 200 column (GE Healthcare) equilibrated with 25 mM Tris–HCl pH 8.0 containing 100 mM or 500 mM NaCl. For the column calibration, besides Ec_RND (45.3 kDa) and Tle1 (96.5 kDa), conalbumin (75 kDa) and aldolase 1 (158 kDa) from the Gel Filtration Calibration Kit HMW (GE Healthcare) were used. The experimental data, with the exception of that for At_NrnC, were used for the linear fit.

Analytical ultracentrifugation experiments were performed using an XL-I analytical ultracentrifuge (Beckman Coulter, Fullerton, CA, United States) equipped with a four-cell An-60 Ti rotor. At_NrnC was diluted to 1 mg/mL in 25 mM Tris–HCl pH 8.0 buffer containing 100 mM NaCl. To determine the influence of metal binding, 5 mM MnCl_2_ was added. The corresponding buffer was used as the reference solution. All samples were centrifuged at 60,000 rpm at 20°C. Analysis of the sedimentation velocity results was performed using the programme Sedfit (Brown and Schuck, 2006).

### Crystallisation and Data Collection

The crystallisation screen for At_NrnC was performed using the vapour diffusion method at 293 K by mixing equal volumes (1 μL/1 μL) of 10 mg/mL At_NrnC protein solution and buffer (0.1 M Tris–HCl, pH 8.0, 1 M (NH_4_)_2_SO_4_). Mn^2+^ binding crystals were prepared by adding 5 mM MnCl_2_ to the reservoir buffer containing the apo crystals and soaking for times ranging from 1 day to 1 week. A mixture of the reservoir solution and 20% (v/v) glycerol was used as the cryoprotectant and the crystals were immersed in liquid nitrogen. X-ray diffraction data were collected at the Shanghai Synchrotron Radiation facility (SSRF) on beamlines BL17U1 and BL19U. The data sets were processed using the HKL-2000 software suite (Otwinowski and Minor, 1997).

### Structure Determination and Analysis

The crystal structures of At_NrnC were determined via molecular replacement using PHASER (McCoy et al., 2007). The manually truncated structure of RNase D from *E. coli* (PDB code: 1YT3) was used as the search model. The models were built using phenix.autobuild and further refined using phenix.refine and COOT (Adams et al., 2010; Emsley et al., 2010). The diffraction data collection and structural refinement statistics are summarised in Table 1.

**Table 1:** Data collection and refinement statistics.

	At_NrnC	At_NrnC-Mn^2+^ (inactive)	At_NrnC-Mn^2+^ (active)
Protein data bank ID	5ZO3	5ZO4	5ZO5
**Data collection**			
Space group	*I*422	*I*422	*I*422
Cell dimensions			
*a*, *b*, *c* (Å)	121.26, 121.26, 133.90	122.01, 122.01, 148.81	121.74, 121.74, 149.97
α, β, γ (°)	90.0, 90.0, 90.0	90.0, 90.0, 90.0	90.0, 90.0, 90.0
Wavelength (Å)	0.9791	0.9791	0.9791
Resolution (Å)	50–1.49 (1.54–1.49)^∗^	50–2.50 (2.59–2.50)	50–2.30 (2.38–2.30)
*R*_merge_ (%)	9.2(37.3)	9.9 (76.3)	8.7 (62.2)
⟨*I*/σ(*I*)⟩	50.45 (8.40)	60.99 (5.56)	28.9 (2.56)
Completeness (%)	99.3 (93.5)	99.8 (100.0)	96.8 (80.2)
Redundancy	14.4 (14.2)	14.3 (14.7)	12.2 (9.2)
**Refinement**			
Resolution (Å)	34.47–1.49	37.32–2.50	47.23–2.30
No. reflections	80479	19726	22848
*R*_work_/*R*_free_ (%)	15.58/17.42	21.33/25.79	18.90/22.45
No. atoms			
Protein	3238	3238	3228
Mn^2+^	0	4	4
Water	677	28	256
*B*-factors			
Protein	12.70	60.34	31.12
Mn^2+^		87.58	68.71
Water	26.14	52.55	33.24
RMS deviations			
Bond lengths (Å)	0.006	0.008	0.002
Bond angles (°)	0.829	1.013	0.411

^∗^*Values in parentheses are for the highest-resolution shell*.

The octameric assembly of At_NrnC and the interfaces of the eight protomers were analysed using PISA (Krissinel, 2015). The central tunnel of the At_NrnC octamer was analysed using HOLE, a programme for the analysis of the pore dimensions of ion channel structural models (Smart et al., 1996). All of the molecular graphics figures were generated using PyMOL.

### At_NrnC Activity Assays

The activity assays toward short single-stranded RNA and DNA substrates were performed using a synthesised RNA 33-mer (5′-GGAGAUAUACAUAUGGCGGUUGCGCAGGACUAU-3′) and a synthesised DNA 33-mer (5′-GGAGATATACATATGGCGGTTGCGCAGGACTAT-3′). After annealing with their complementary fragments, these substrates were used for the assays. The activity assays toward long double-stranded DNA were performed using a PCR-amplified 1300-bp fragment. The reaction buffer for was 50 mM Tris–HCl pH 8.8 containing 5 mM MnCl_2_. The activity assays were performed in a total volume of 25 μL using 2.5 μL of a 1 mg/mL nuclease solution and 10 μL of a 20 μM RNA or DNA substrate solution (100 μg/mL for the 1300-bp fragment) and incubated for 30 min at 37°C. The reactions were terminated by the addition of EDTA to a final concentration of 25 mM, and 5 μL of proteinase K (Takara) was then added followed by incubation at 50°C for 30 min to degrade At_NrnC. The reaction mixtures were loaded onto agarose gels and electrophoresis was performed at 160 V for 12 min (20 min for the 1300-bp fragment). The gels were stained with ethidium bromide.

### Accession Numbers

Atomic coordinates and structure factors for the reported crystal structures have been deposited with the Protein Data Bank under accession numbers 5ZO3, 5ZO4, and 5ZO5.

## Author Contributions

LG and ZY designed the experiments. ZY and FG performed the experiments, collected the data, and determined the structures. ZY, FG, KY, and LG analysed the data. ZY and LG wrote the manuscript. All authors read and approved the manuscript.

## Conflict of Interest Statement

The authors declare that the research was conducted in the absence of any commercial or financial relationships that could be construed as a potential conflict of interest.
