# Supplementary material for: NrnC, an RNase D-Like Protein From Agrobacterium, Is a Novel Octameric Nuclease That Specifically Degrades dsDNA but Leaves dsRNA Intact
Source: Front Microbiol. 2019 Jan 7;9:3230. doi: 10.3389/fmicb.2018.03230 (PMC6330322; doi:10.3389/fmicb.2018.03230)
Supplement: Supplementary file 1 [file Data_Sheet_1.docx]

***Supplementary Material***

**NrnC, an RNase D-like protein from Agrobacteria is a novel octameric nuclease which specifically degraded dsDNA but does not harm dsRNA**

***Zenglin Yuan, Fei Gao, Kun Yin and Lichuan Gu^*^***

*** Correspondence:** Lichuan Gu: lcgu@sdu.edu.cn

**Supplementary Figures**

**
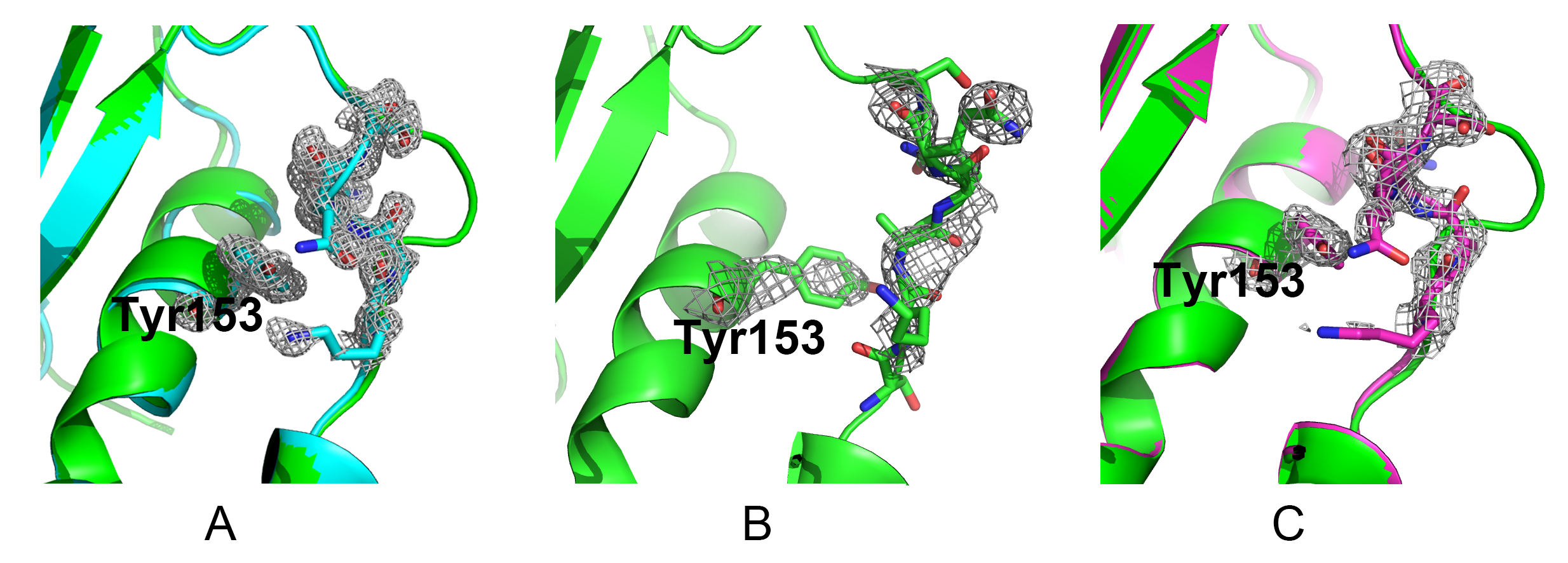
**

**FIGURE S1. 2Fo-Fc electron density map of Tyr153 and the loop Lys134 to Ser138.** All maps are contoured at 1.0 σ. Although the electron density for the side chains of these residues is not completely elucidated, still the backbone of the K134-S138 peptide and the direction of the side chain of the Y153 can be traced. **(A)** The apo At_NrnC is shown as cartoon in cyan and the inactive Mn^2+^-bound At_NrnC is shown as cartoon in green. Y153 and the K134-S138 loop of apo At_NrnC are shown as sticks, and the corresponding density map is shown as well. **(B)** The inactive Mn^2+^-bound At_NrnC is shown as cartoon in green. Y153 and the K134-S138 loop are shown as sticks and the corresponding density map is shown. **(C)** The active Mn^2+^-bound At_NrnC is shown as cartoon in magenta and the inactive Mn^2+^-bound At_NrnC is shown as cartoon in green. Y153 and the K134-S138 loop of active Mn^2+^-bound At_NrnC are shown as sticks and the corresponding density map is also shown.

**
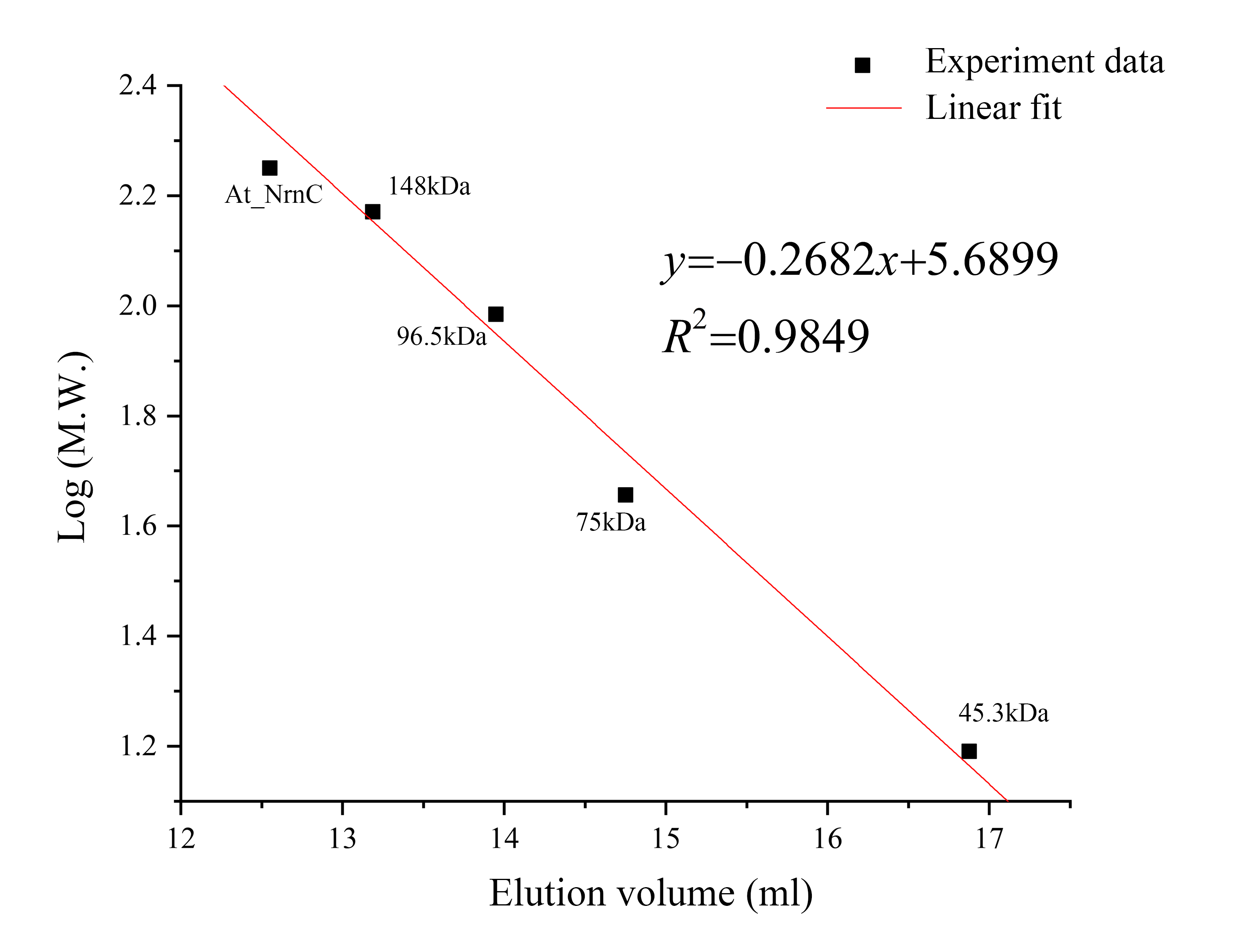
**

**FIGURE S2. Column calibration.** Besides Ec_RND (45.3 kDa) and Tle1 (96.5 kDa), conalbumin (75 kDa) and aldolase1 (158 kDa) from the Gel Filtration Calibration Kit HMW (GE Healthcare) have been used. The experimental data, with the exception of that for At_NrnC, were used for the linear fit.


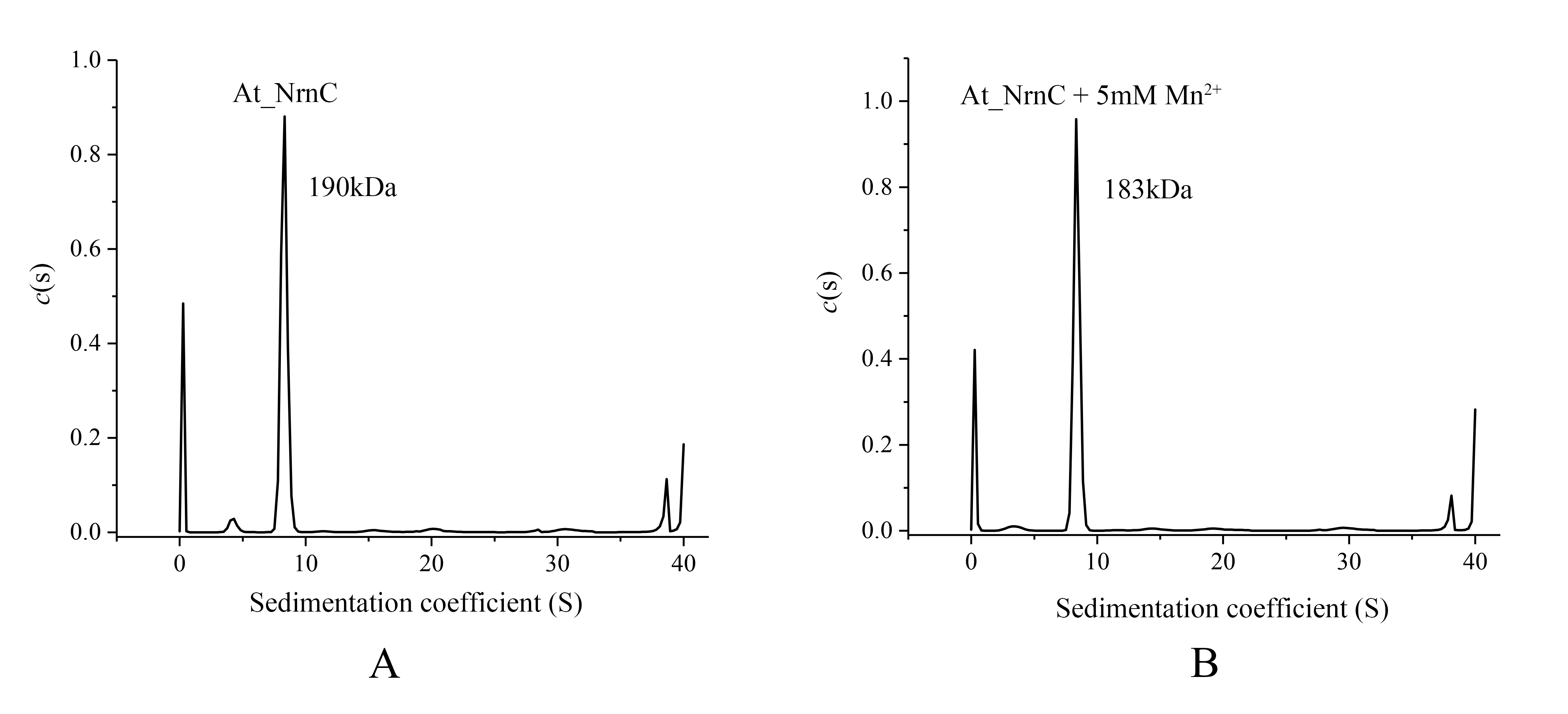


**FIGURE S3. Analytical ultracentrifugation analysis. (A)** The molecular weight of At_NrnC is calculated as 190 kDa in solution. **(B)** With 5 mM MnCl_2_, the molecular weight of At_NrnC is calculated as 183 kDa in solution.


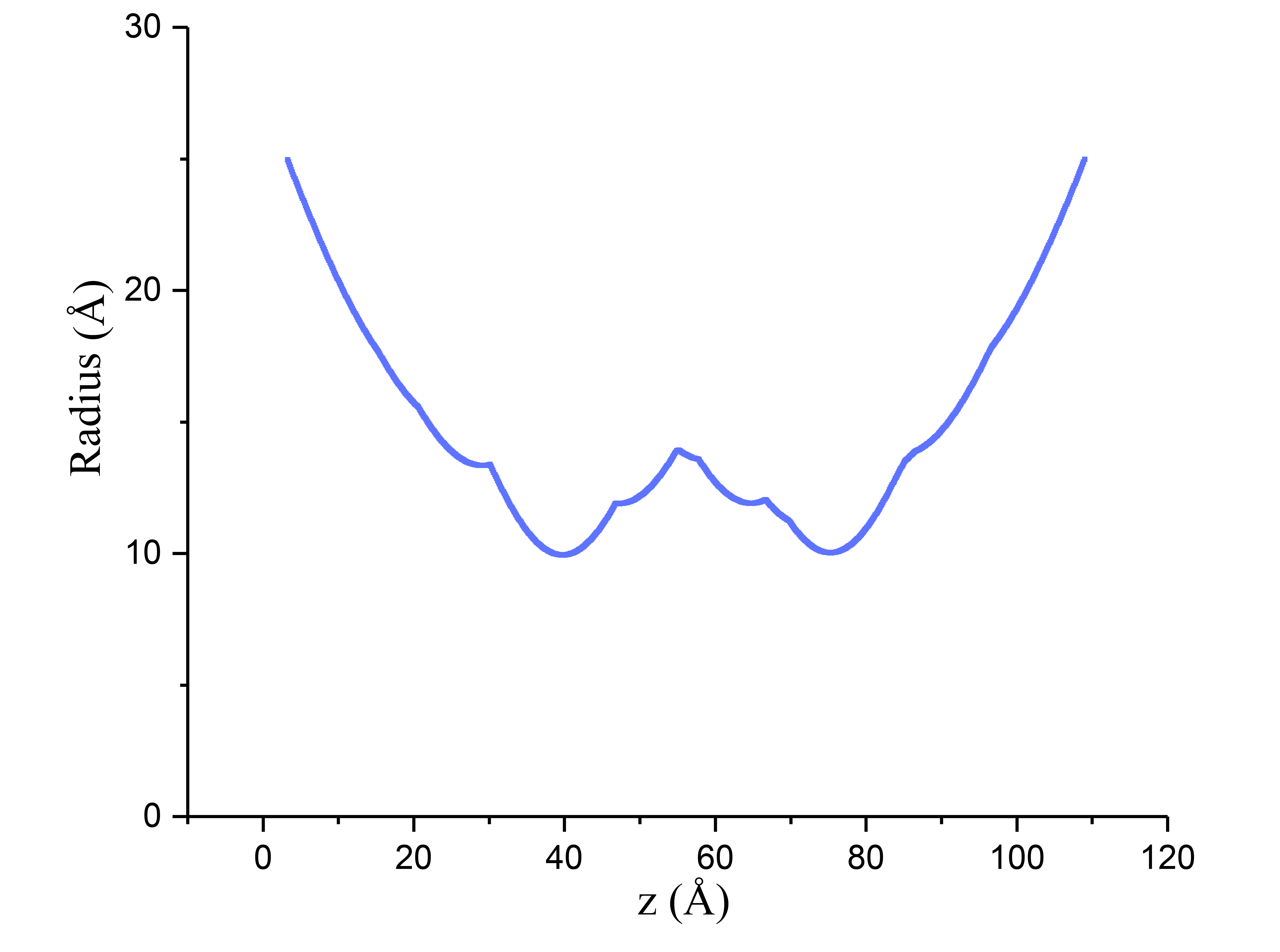


**FIGURE S4. Pore-Radius Profiles of At_NrnC.** The radius is 10 Å for the narrowest part, which corresponds to a diameter of 20 Å.
